# Acute Renal Failure and Volume Overload Syndrome Secondary to a Femorofemoral Arteriovenous Fistula Angioplasty in a Kidney Transplant Recipient

**DOI:** 10.1155/2013/197524

**Published:** 2013-02-26

**Authors:** Dominique Bertrand, Geoffroy Desbuissons, Nicolas Pallet, Albane Sartorius, Christophe Legendre, Marie-France Mamzer, Rebecca Sberro Soussan

**Affiliations:** Service de Transplantation Rénale et Soins Intensifs, Hôpital Necker, Assistance Publique Hôpitaux de Paris, Université Paris Descartes, 75015 Paris, France

## Abstract

Experimental and clinical studies analyzing the impact of AVF on cardiovascular and renal parameters, as well as outcomes, in kidney transplant recipients are lacking. On the other hand, it is not known whether AVF ligation after transplantation modifies hemodynamic parameters and kidney function. We report a case of a renal transplant recipient who developed an acute congestive heart failure accompanied by renal failure, which were triggered by femorofemoral AVF angioplasty. Prompt AVF ligation rapidly reversed clinical symptoms and normalized cardiac and renal functions. This paper illustrates the potential deleterious consequences of high-output AVF after kidney transplantation and raises considerations regarding the impact of the fistula on cardiac status and kidney function after kidney transplantation and, consequently, the management AVF after transplantation.

## 1. Introduction

Arteriovenous fistula (AVF) is the preferred access for hemodialysis, because of its longevity and resistance to infections [1]. However, large left-to-right shunts generate hemodynamic disturbances that may be deleterious for the cardiovascular system [2]. AVF induces a drop in peripheral vascular resistance, which leads to a compensatory increase in cardiac output. Sympathetic nervous system is activated, leading to an increasing pulse rate, stroke volume, and contractility.

If high-output cardiac failure is underrecognized during hemodialysis, because of the absence of obvious volume overload, the deleterious cardiovascular effect of AVF can occur after kidney transplantation. However, experimental and clinical studies analyzing the impact of AVF on cardiovascular and renal parameters, as well as outcomes, in kidney transplant recipients are lacking.

On the other hand, it is not known whether AVF ligation after transplantation modifies hemodynamic parameters and kidney function. There is no consensus regarding AVF management after kidney transplantation, and fistula are usually ligated according to the patient wishes and if the clinical situation is controlled.

Here we report a case of a renal transplant recipient with a femorofemoral AVF who developed an acute congestive heart failure accompanied by renal failure, which were triggered by AVF angioplasty. Prompt AVF ligation rapidly reversed clinical symptoms and normalized cardiac and renal functions.

## 2. Case

A 51-year-old Caucasian woman was admitted to our department in April 2011 for a third kidney transplantation. Focal segmental glomerulosclerosis (FSGS) has been diagnosed in 1973, and the patient reached end-stage renal failure in 1982. He received two kidney allografts, in 1983 and 1993, which both failed as a consequence of early recurrence of FSGS. Since 2002, hemodialysis was performed on a left femorofemoral fistula, because it was impossible to create new upper arm fistula after multiple episodes of thrombosis.

The third kidney transplantation was performed in April 2011 on the right iliac fossa. The patient was not immunized, and cold ischemia time was 23 hours. The patient received thymoglobulin as an induction therapy, with mycophenolate mofetil daily, and tacrolimus and corticosteroids as maintenance therapy. After an initial delayed graft function, renal function improved, and on day 10 serum creatinine was 155 *µ*mol/L.

A rise of serum creatinine at 180 *µ*mol/L on day 10 led to the initial diagnosis of moderate acute humoral rejection (g1, cpt1, C4d 3, and acute tubular necrosis), despite the absence of donor specific antibodies. Serum creatinine improved after an increase of corticosteroids doses and plasma exchange therapy.

The patient was discharged 3 weeks after transplantation with serum creatinine at 124 *µ*mol/L.

A phlebography with carbon dioxide followed by an angioplasty of the femorofemoral fistula was performed 3 weeks after discharge because of a stenosis diagnosed before the transplantation, leading to decreased blood flow and difficulties in cannulation.

Three days after angioplasty, the patient was readmitted for volume overload syndrome with peripheral oedema and shortness of breath.

On admission, the patient was afebrile, its blood pressure was 118/66 mmHg, and its pulse rate 83/min. A weight gain of 4 kg was noticed. His respiratory rate was 27/min, and oxygen saturation was 96% on ambient air. Physical examination revealed crackles in the two lung fields, jugular venous distension, and pitting peripheral oedema.

Medications included prednisone, mycophenolate mofetil, tacrolimus, esomeprazole, trimethoprim/sulfamethoxazole, and furosemide.

Blood urea nitrogen was 18 mmol/L, serum creatinine 200 *µ*mol/L, sodium 135 mmol/L, troponin < 0,01 ng/mL, brain natriuretic peptide 500 ng/L (normal < 100 ng/L), hemoglobin 10 g/dL, natriuresis 10 mmol/L, and kaliuresis 55 mmol/L. There was no significant proteinuria. Liver enzymes were slightly elevated. Electrocardiogram was not modified, and chest radiography showed bilateral alveolointerstitial syndrome.

Intravenous diuretics therapy with high dose of furosemide, hydrochlorothiazide, and spironolactone was administered, but did not lead to a clinical improvement.

Echocardiograms showed a moderate left ventricular hypertrophy with normal function and no sign for right heart failure with normal pulmonary arterial pressure.

A duplex ultrasound revealed an increased blood flow of the AVF, estimated at 1400 mL/min. Allograft vascularisation was not influenced by the AVF. There was no evidence for a renal artery stenosis or iliac artery stenosis, which was confirmed by a nuclear magnetic resonance imaging.

A kidney allograft biopsy was performed which showed that there was no sign of antibody mediated rejection (g0, cpt0, and C4d score = 1).

Despite normal echocardiogram, a right heart catheterization was performed. The mean pulmonary arterial pressure was 44 mmHg, cardiac index was 3,1 L/min/m^2^, and pulmonary capillary wedge pressure was 30 mmHg. Systemic arterial resistance was decreased. The diagnosis was heart failure triggered by high blood flow arteriovenous fistula.

This was confirmed by AVF ligature, which led to an increase of urine output and natriuresis, kidney allograft function improvement (Table 1), and loss of weight and peripheral oedema.

## 3. Discussion

This paper illustrates the potential acute deleterious consequences of AVF after kidney transplantation and raises considerations regarding the impact of the fistula on cardiac status and kidney function after kidney transplantation and, consequently, the management AVF after transplantation.

The long-term consequences of AVF on cardiac parameters of renal transplant recipients have been relatively well studies, but the clinical outcomes are less understood. Echocardiographic abnormalities have been reported to be associated with AVF in renal transplant patients, and one study showed that, in stable asymptomatic kidney transplant patients, a functioning AVF may have significant impact on cardiac mass, cardiac index, and left ventricular dimensions [3]. The impact of AVF-induced hemodynamic changes on patients and allograft outcomes are unknown. However, AVF increases pulmonary flow, arterial pressure, and pulmonary hypertension before transplantation and correlates with patient survival after kidney transplantation. This suggests that a noninvasive screening for pulmonary hypertension before transplantation could be useful, especially if an AVF is actually functioning. The closure of large and symptomatic AVF in renal transplant recipients is associated with long-term regression of left ventricular hypertrophy [4], but the clinical impact of closure of asymptomatic AVF in renal transplant recipients remains to be determined.

Beside cardiac remodeling and functions, AVF can negatively influence allograft function. AVF may directly induce renal failure by a steal syndrome, as reported by Kuwertz-Broking et al. [5] in 1988. AVF can lead to the hypoperfusion of the allograft, which induces a reversible decrease of kidney function after ligation. The systemic hemodynamic changes induced by AVF may also induce renal allograft failure. Pulmonary hypertension with consecutive right heart failure is associated with tricuspid regurgitation, inferior cava vein congestion, and subsequent iliac congestion, which may have deleterious effects on kidney allograft perfusion because of reduced venous efflux. The first case [6] has been described as a renal transplant recipient with cardiac and renal failure, complicating an arteriovenous fistula. More recently, Meier et al. reported four patients with dilated right ventricle after kidney transplantation [7]. Duplex ultrasound detected high-flow AVF, and right heart catheterization confirmed pulmonary hypertension and an elevated cardiac index, and both decreased after AVF obliteration. Thus, AVF were ligated, leading to a spectacular improvement of kidney allograft function, illustrating the deleterious, but reversible, impact of right heart failure caused by AVF.

The case reported here illustrates for the first time the acute deleterious consequences after an abrupt increase of AVF on both cardiac and kidney functions. This “experimental” increase of AVFblood flow after angioplasty leads to an acute pulmonary hypertension with cardiac heart failure with subnormal cardiac index. The acute pulmonary hypertension and right heart failure lead to a congestive venous status and an acute kidney failure, which was reversible after AVF ligature.

After kidney transplantation, overload syndrome can decompensate with acute renal failure and right heart failure. In this situation, AVF may to be closed. As a preventive strategy, AVF may also be ligated, if the kidney transplant recipient has a medical history of heart failure or coronary arterial disease, even stable, and if kidney allograft function is stable.

In conclusion, the case presented here demonstrates that high-output AVF may profoundly and abruptly induce hemodynamic changes, leading to a congestive heart failure and kidney allograft dysfunction. In case of unexplained volume overload syndrome after kidney transplantation, the implication of AVF has to be demonstrated, even with a right heart catheterization.

## Figures and Tables

**Table 1:** 

	Day −1	Day 0	Day 1	Day 2	Day 3	Day 4
Serum creatinine (*μ*mol/L)	270		230	170	150	133
Natriuresis (mmol/L)	<10		28	55	30	32
Kaliuresis (mmol/L)	36	AVF closure	42	29	15	15
Weight (kg)	65		64,1	62,6	61,8	60
Furosemide dose (mg)	375		250	125	100	60
